# Supplementary material for: Effect of biannual azithromycin distribution on antibody responses to malaria, bacterial, and protozoan pathogens in Niger
Source: Nat Commun. 2022 Feb 21;13:976. doi: 10.1038/s41467-022-28565-5 (PMC8861117; doi:10.1038/s41467-022-28565-5)
Supplement: Supplementary file 1 — Supplementary Information [file 41467_2022_28565_MOESM1_ESM.pdf]

Effect of biannual azithromycin distribution on antibody responses to malaria, bacterial, and protozoan pathogens in Niger

Ahmed M. Arzika, Ramatou Maliki, E. Brook Goodhew, Eric Rogier, Jeffrey W. Priest, Elodie Lebas, Kieran S. O'Brien, Victoria Le, Catherine E. Oldenburg, Thuy Doan, Travis C. Porco, Jeremy D. Keenan, Thomas M. Lietman, Diana L. Martin, Benjamin F. Arnold, MORDOR-Niger Study Group

Supplementary Information Materials

MORDOR-Niger Study Group

Authors are ordered by institution and then alphabetically by surname.

University of California, San Francisco, San Francisco, CA, USA
Benjamin F Arnold [4,5], Catherine Cook [4], Sun Y Cotter [7], Thuy Doan [4,5], Dionna M Wittberg [4], Jeremy D Keenan [4,5], Elodie Lebas [4], Thomas M Lietman [4,5,6], Kieran S O'Brien [4,5], Catherine E Oldenburg [4,5,6], Travis C Porco [4,5,6], Kathryn J Ray [4], Philip J Rosenthal [7,8], George W Rutherford [6,7], Benjamin Vanderschelden [4], Nicole E Varnado [4], Lina Zhong [4], Zhaoxia Zhou [4]

The Carter Center Niger, Niamey, Niger
Ahmed M Arzika [1], Sanoussi Elh Adamou [1], Nana Fatima Galo [1], Fatima Ibrahim [1], Salissou Kane [1], Mariama Kiemago [1], Ramatou Maliki [1]

The Carter Center, Atlanta, GA, USA
E Kelly Callahan [9], Aisha E Stewart [9]

Programme National de Santé Oculaire, Niamey, Niger
Amza Abdou [10], Nassirou Beido [10], Boubacar Kadri [10]

Johns Hopkins University, Baltimore, MD, USA
Jerusha Weaver [11], Sheila K West [11]

International Trachoma Initiative, Decatur, GA, USA
Paul M Emerson [12]

[1] The Carter Center Niger, Niamey, Niger. [4] Francis I. Proctor Foundation, University of California, San Francisco, CA, USA. [5] Department of Ophthalmology, University of California, San Francisco, CA, USA. [6] Department of Epidemiology and Biostatistics, University of California, San Francisco, CA, USA. [7] Global Health Sciences, University of California, San Francisco, CA, USA. [8] Department of Medicine, University of California, San Francisco, CA, USA. [9] The Carter Center, Atlanta, GA, USA. [10] Programme National de Santé Oculaire, Niamey, Niger. [11] Johns Hopkins University, Baltimore, MD, USA. [12] International Trachoma Initiative, Decatur, GA, USA.

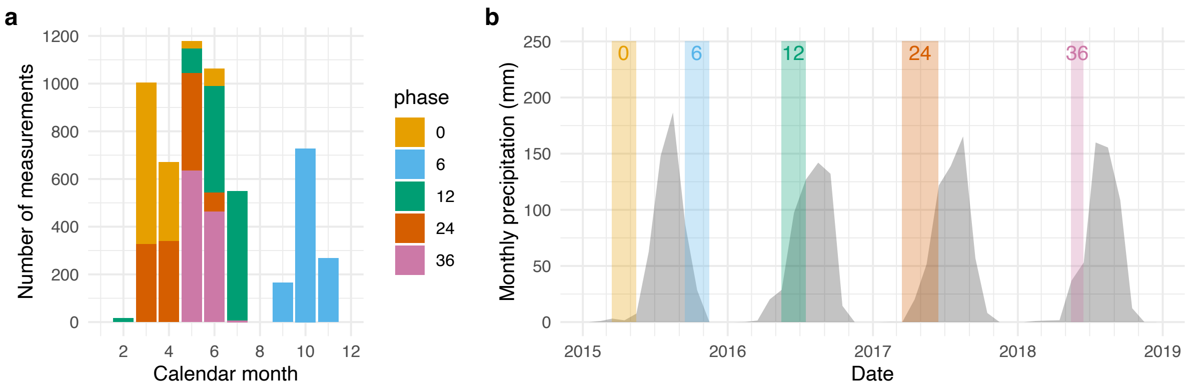

Supplementary Figure 1. **Dried blood spot measurement timing and monthly rainfall in the MORDOR Niger trial**. **a** Specimens collected by calendar month and study phase (months since baseline), showing that most blood spot specimens were collected March-July, except those in phase 6. **b** Monthly precipitation in study communities. Colored periods show the timing of dried blood spot specimen collection for each study phase. Created with notebook https://osf.io/pem3z .

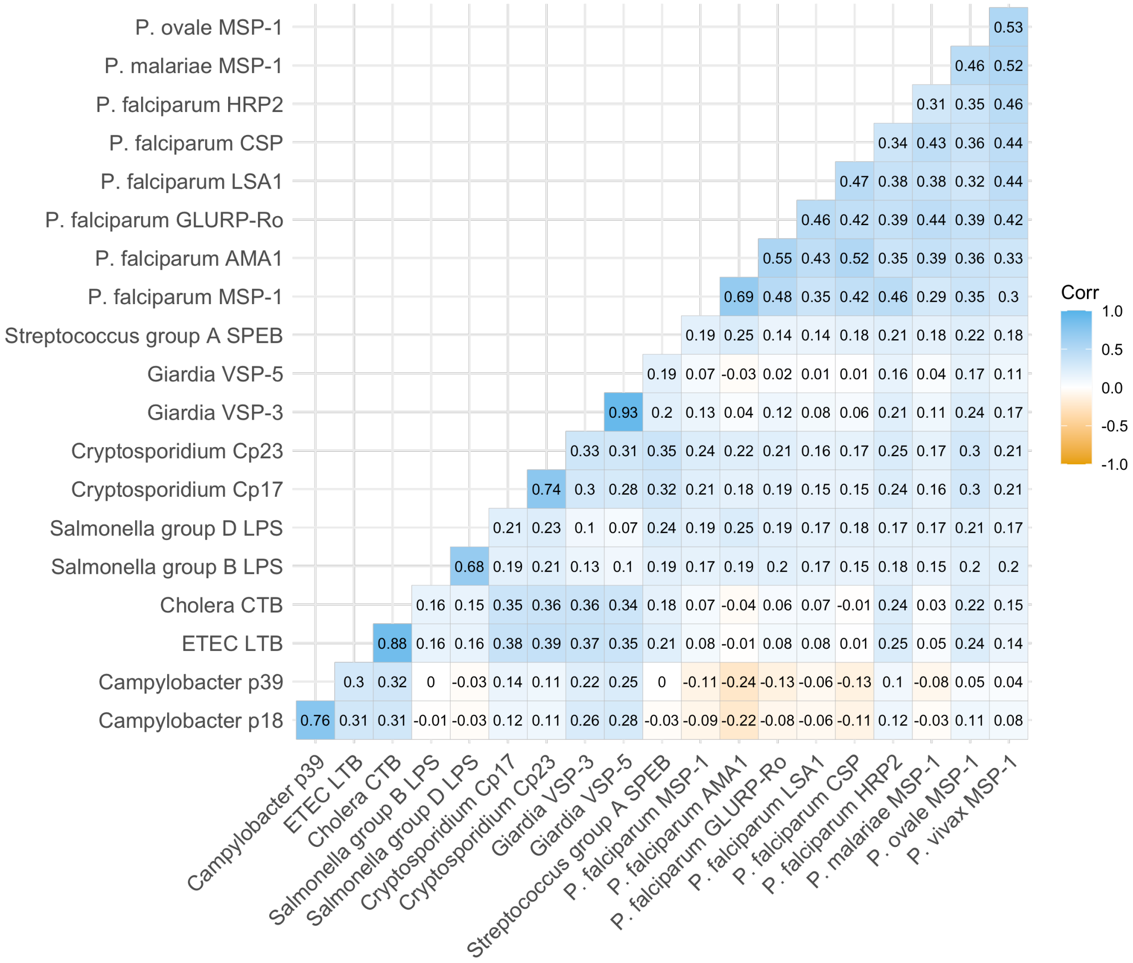

Supplementary Figure 2. Pairwise correlation between antibody responses to antigens included in the multiplex assay. Correlation estimated from 5,642 samples among children ages 1-59 months in Niger using log_{10}-transformed median fluorescence intensity minus background (MFI-bg) IgG responses measured on the Luminex platform. Cells are colored by the strength of pair-wise correlation (printed). Created with notebook https://osf.io/t9wjk/.

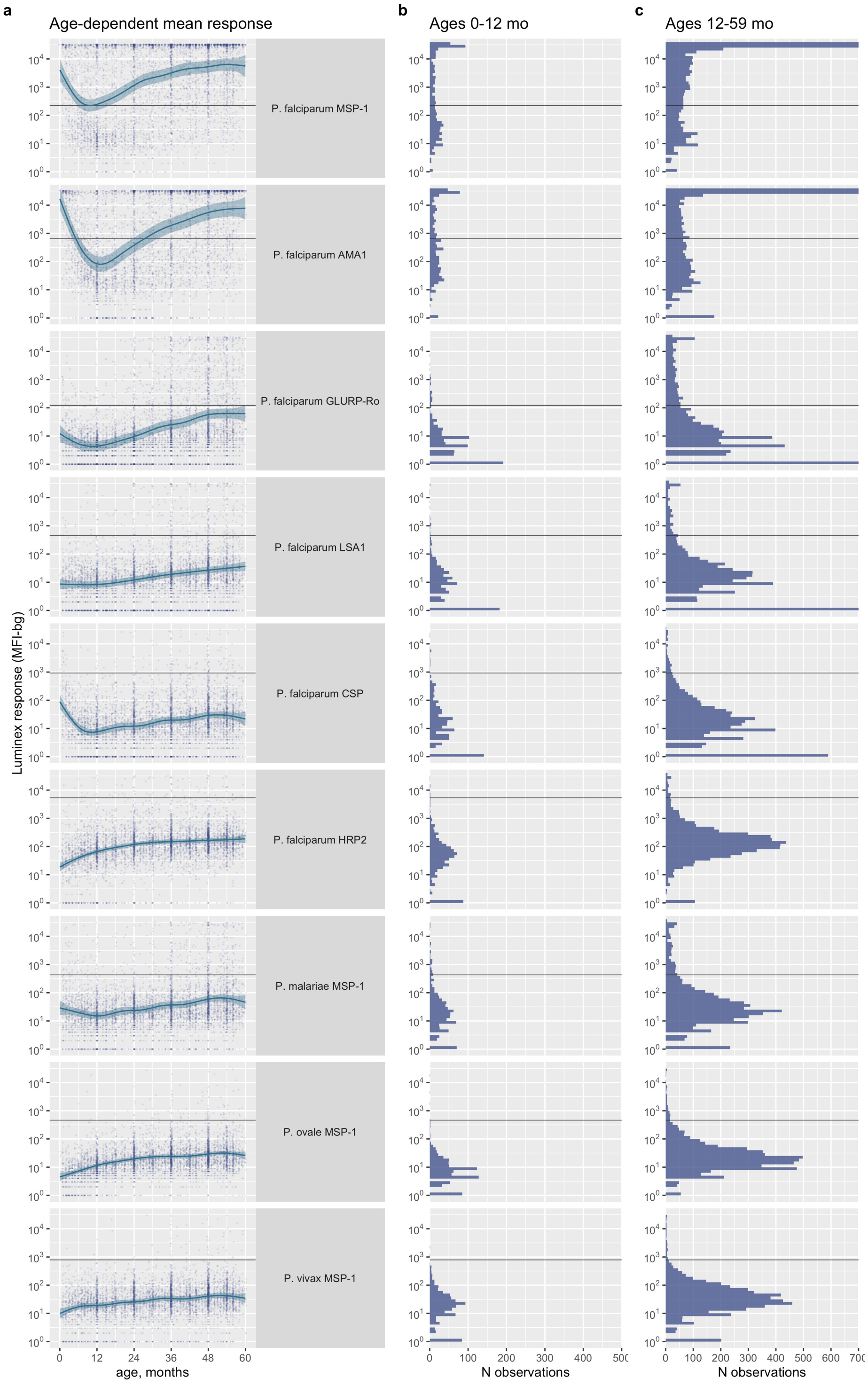

Supplementary Figure 3. Malarial antibody responses among children ages 0-59 months in MORDOR Niger. a Age dependent mean response estimated with cubic splines (lines), shaded bands are simultaneous 95% confidence intervals. Distribution of antibody response among children **b** ages 0-12 months, and **c** ages 12.1 to 59 months. Horizontal lines mark the seropositivity cutoffs derived from panels of negative sera. Created with notebook https://osf.io/vqtbm.

Supplementary Figure 4. **Malarial IgG antibody responses by age and treatment group.** Estimates derived from 5,642 measurements among children ages 1-59 months in the MORDOR Niger trial, 2015-2018 **a** Antigen-specific geometric mean IgG responses (MFI-bg) by age and treatment group. **b** Antigen-specific seroprevalence by age and treatment group. Group means(lines) estimated using semiparametric cubic splines. Shaded bands indicate simultaneous 95% confidence intervals. Shaded area in each panel indicates the age range included in analyses (n = 3,860 children ages 12-59 months). Created with notebook https://osf.io/smwbn .

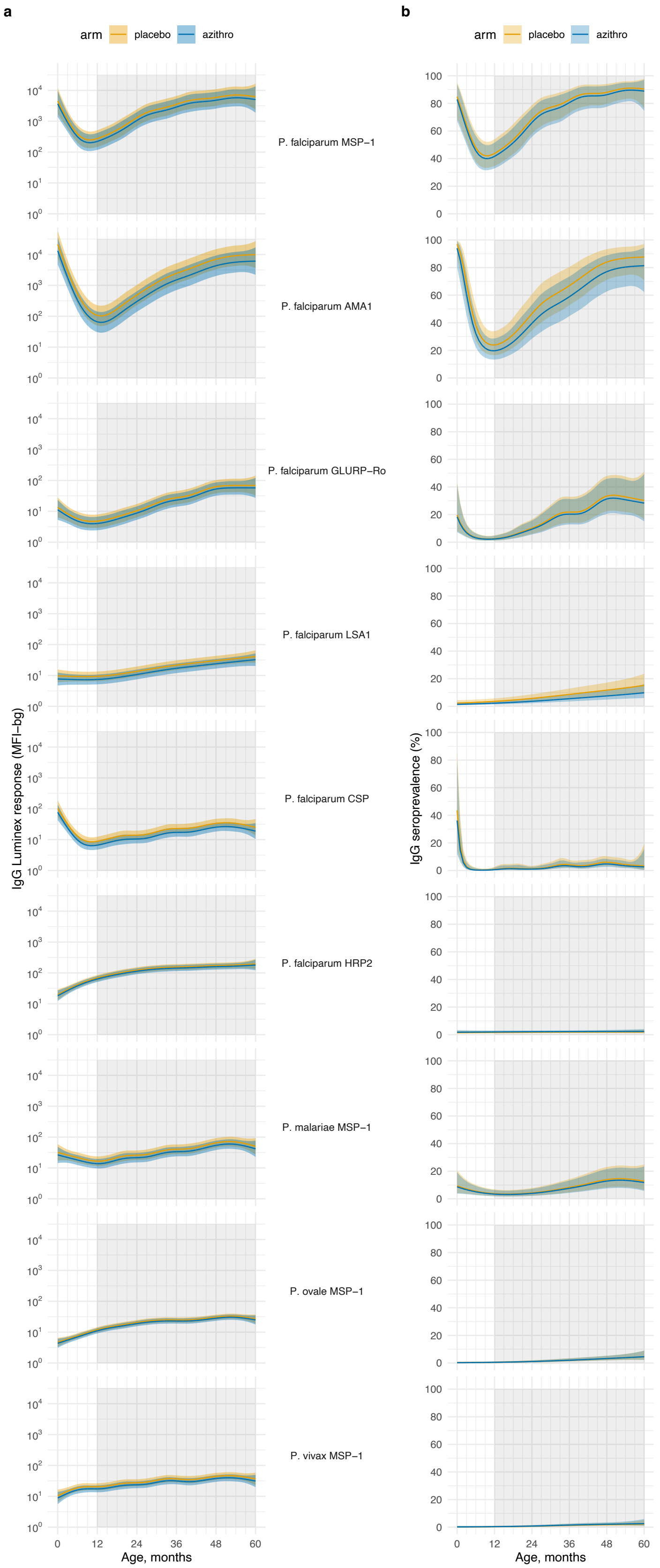

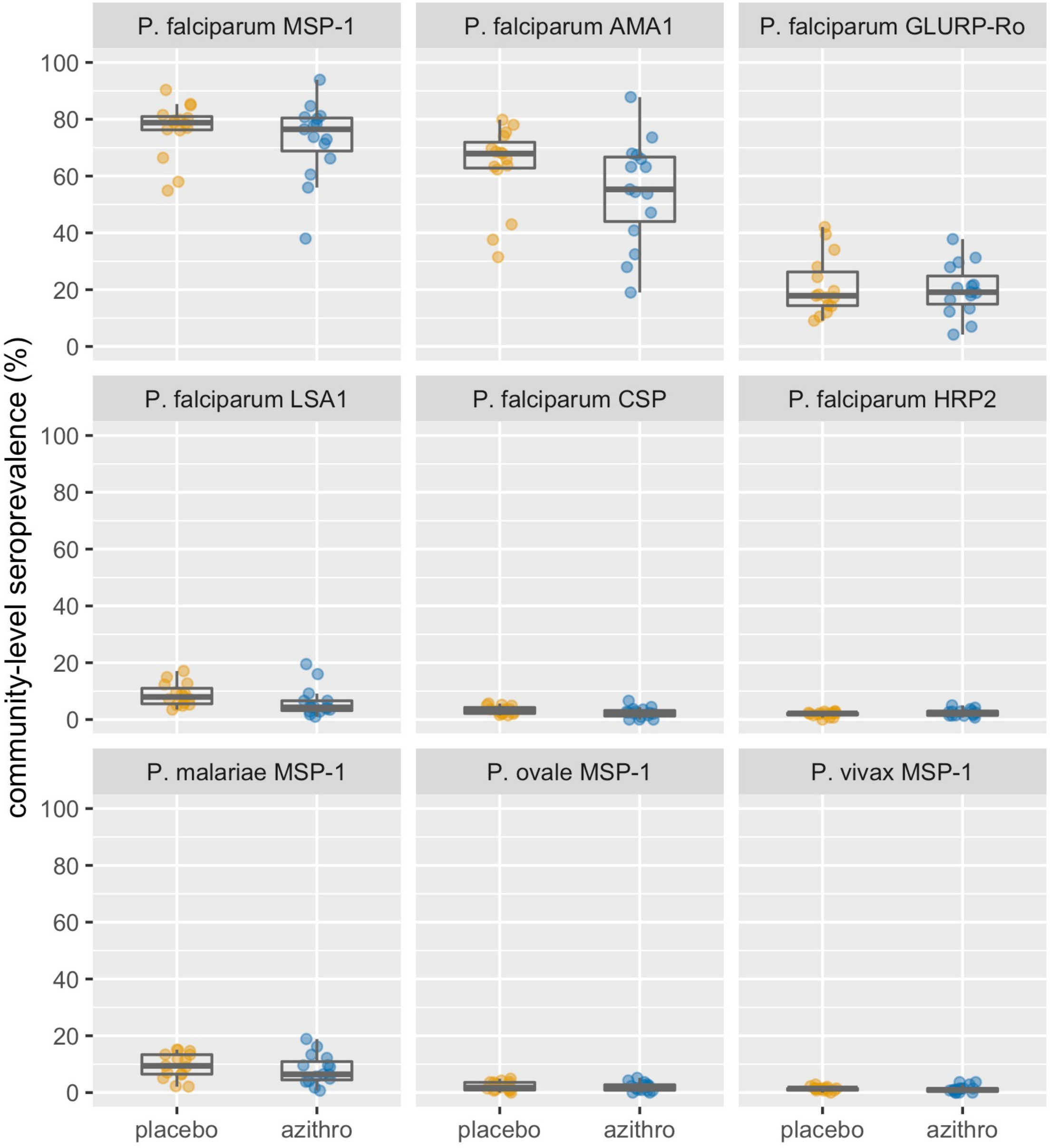

Supplementary Figure 5. Community level IgG seroprevalence for malarial antigens. Estimates derived from 3,860 children ages 12-59 months in the MORDOR Niger trial, 2015-2018. Box plots indicate median (middle line) and 25th, 75th percentile (box) and 1.5 times the interquartile range (whiskers) for the 15 communities (points) in each of the groups (placebo, azithromycin). Created with notebook https://osf.io/b2v3r .

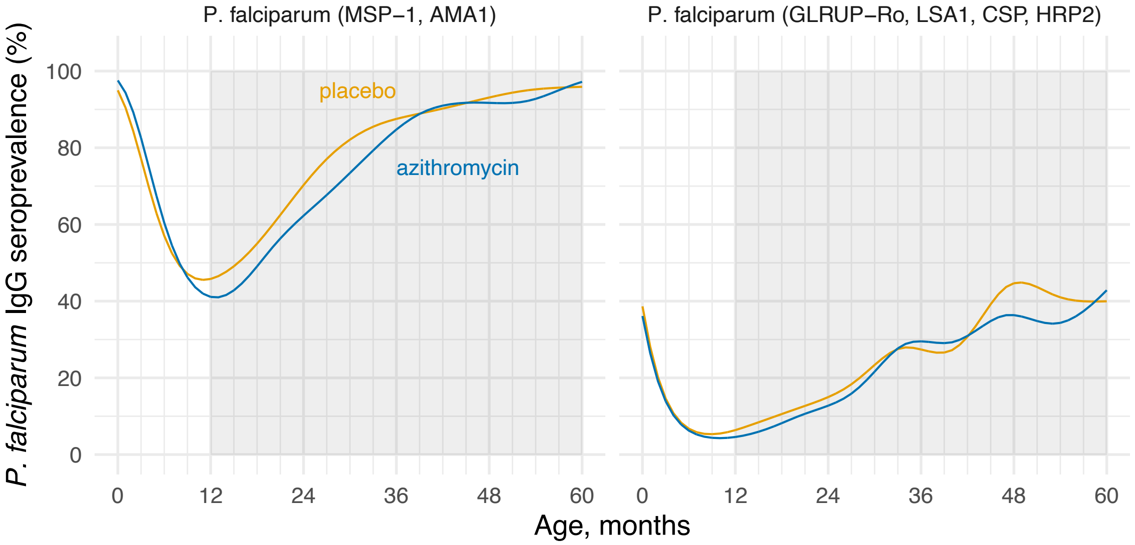

Supplementary Figure 6. ***Plasmodium falciparum* IgG seroprevalence by age and treatment group for different antigen sets.** Seroprevalence by age and treatment group to longer-lived IgG responses (MSP-1, AMA1) and shorter-lived IgG responses (GLRUP-Ro, LSA1, CSP, HRP2) among children in the MORDOR Niger trial. Group means (lines) estimated using semiparametric cubic splines. Shaded area indicates age range included in analyses (n = 3,860 children ages 12-59 months).

Following methods of the primary analysis, seroprevalence was not statistically different between groups based longer-lived IgG responses (azithromycin 75% vs placebo 80%, difference: –5%, 95% CI –13% to 2%) or shorter-lived responses (25% vs 28%, difference: –2%, 95% CI –10% to 5%). Estimation of the relative hazard from age-structured seroprevalence was also similar between the two groups of antigen: hazard ratio (HR)=0.88, 95% CI 0.61 to 1.26 for longer lived antigens, HR = 0.87, 95% CI 0.58 to 1.32 for shorter-lived antigens. Created with notebook https://osf.io/37ybm, which includes detailed point estimates.

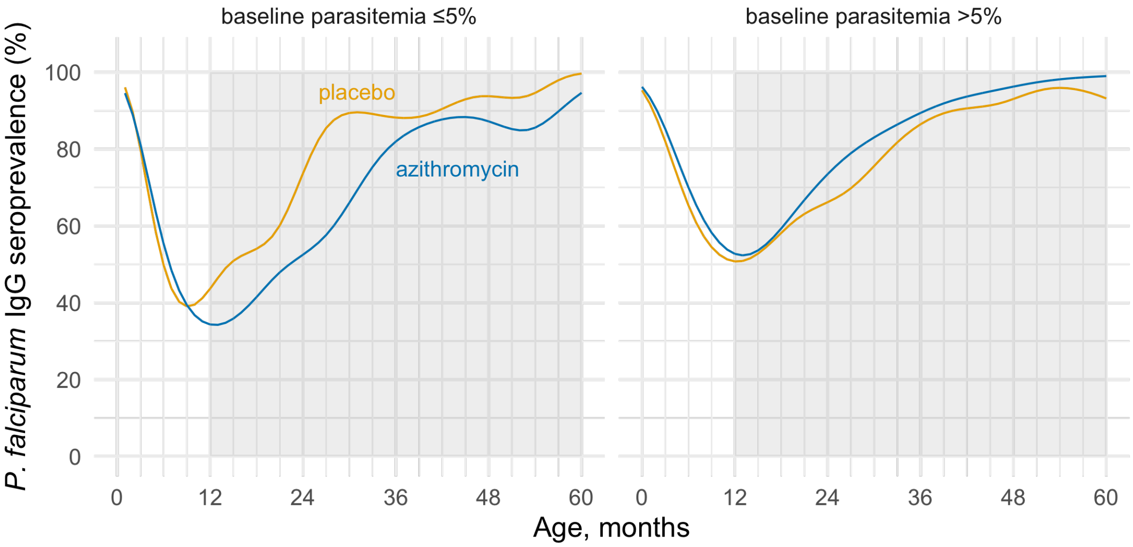

Supplementary Figure 7. ***Plasmodium falciparum* IgG seroprevalence by age, treatment group, and baseline malaria parasitemia**. Seroprevalence by age and treatment group among 17 communities with baseline parasitemia ≤5% (n=2,174 children 12-59 months) and 13 communities with baseline parasitemia >5% (n=1,686 children 12-59 months) in the MORDOR Niger trial. Group means (lines) estimated using semiparametric cubic splines. Shaded area indicates age range included in analyses (n = 3,860 children ages 12-59 months).

Following methods of the primary analysis, seroprevalence was not statistically different in either the low parasitemia subgroup (azithromycin 70% vs placebo 81%, difference: –11%, 95% CI –22% to 2%) or the higher parasitemia subgroup (82% vs 79%, difference: 3%, 95% CI –4% to 12%), with formal test of interaction on the additive scale *P*=0.06. Estimation of the relative hazard from age-structured seroprevalence was consistent with the seroprevalence results: hazard ratio (HR) = 0.68, 95% CI 0.43 to 1.07 for baseline parasitemia ≤5%, HR = 1.21, 95% CI 0.76 to 1.92 baseline parasitemia >5%. Created with notebook https://osf.io/pmg4a/.

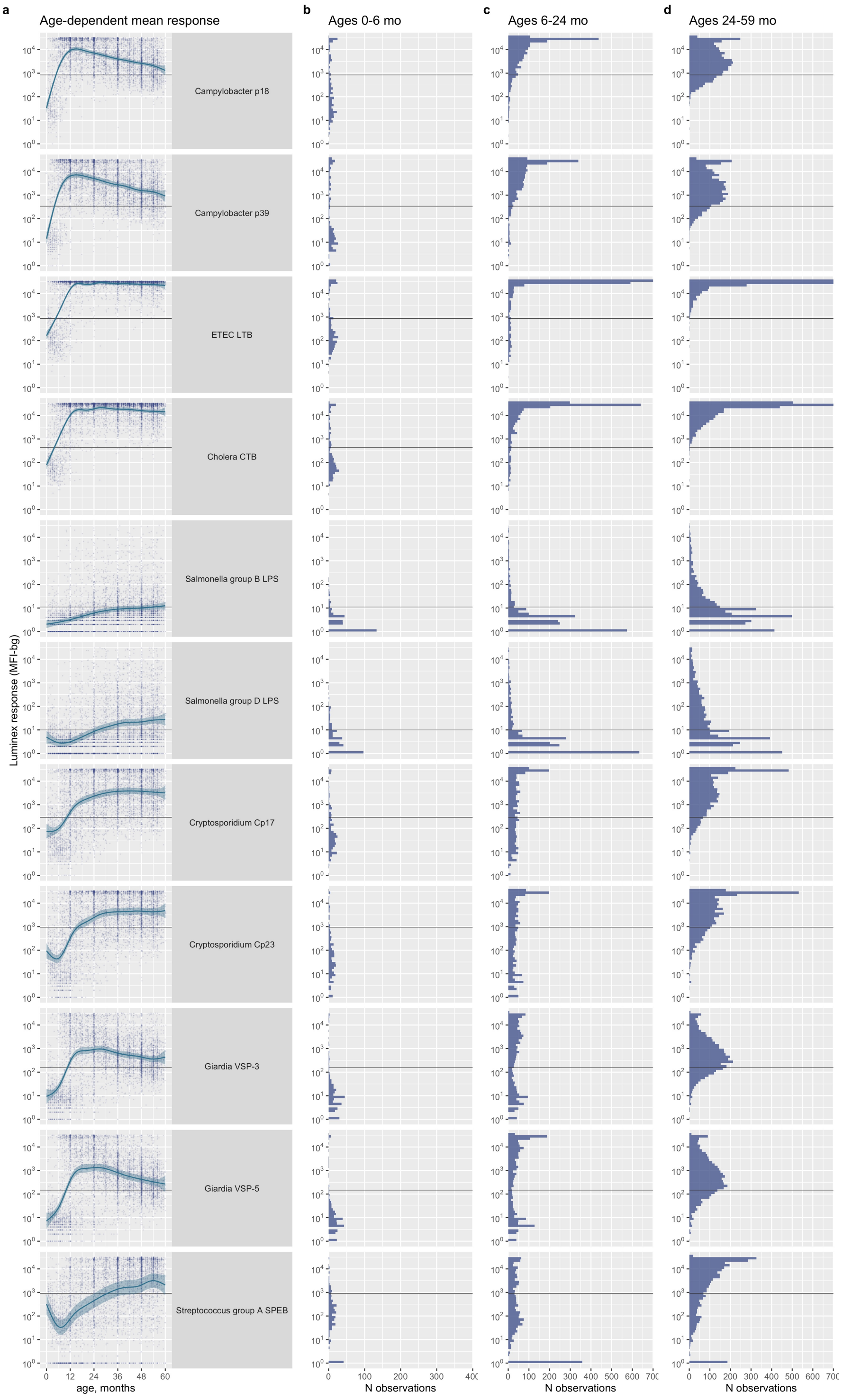

Supplementary Figure 8. **Antibody responses to bacteria and protozoan pathogens among children ages 0-59 months in MORDOR Niger**. **a** Age dependent mean response estimated with cubic splines (lines), shaded bands are simultaneous 95% confidence intervals. Distribution of antibody response among children **b** ages 0-6 months**, c** ages 6.1 to 24 months, and **d** ages 24.1 to 59 months. Horizontal lines mark the seropositivity cutoffs derived through ROC curves (*Cryptosporidium* sp., *Giardia* sp.) or using the mean plus 3 times the standard deviation among presumed unexposed children (others, described in Methods). Created with notebook https://osf.io/vqtbm.

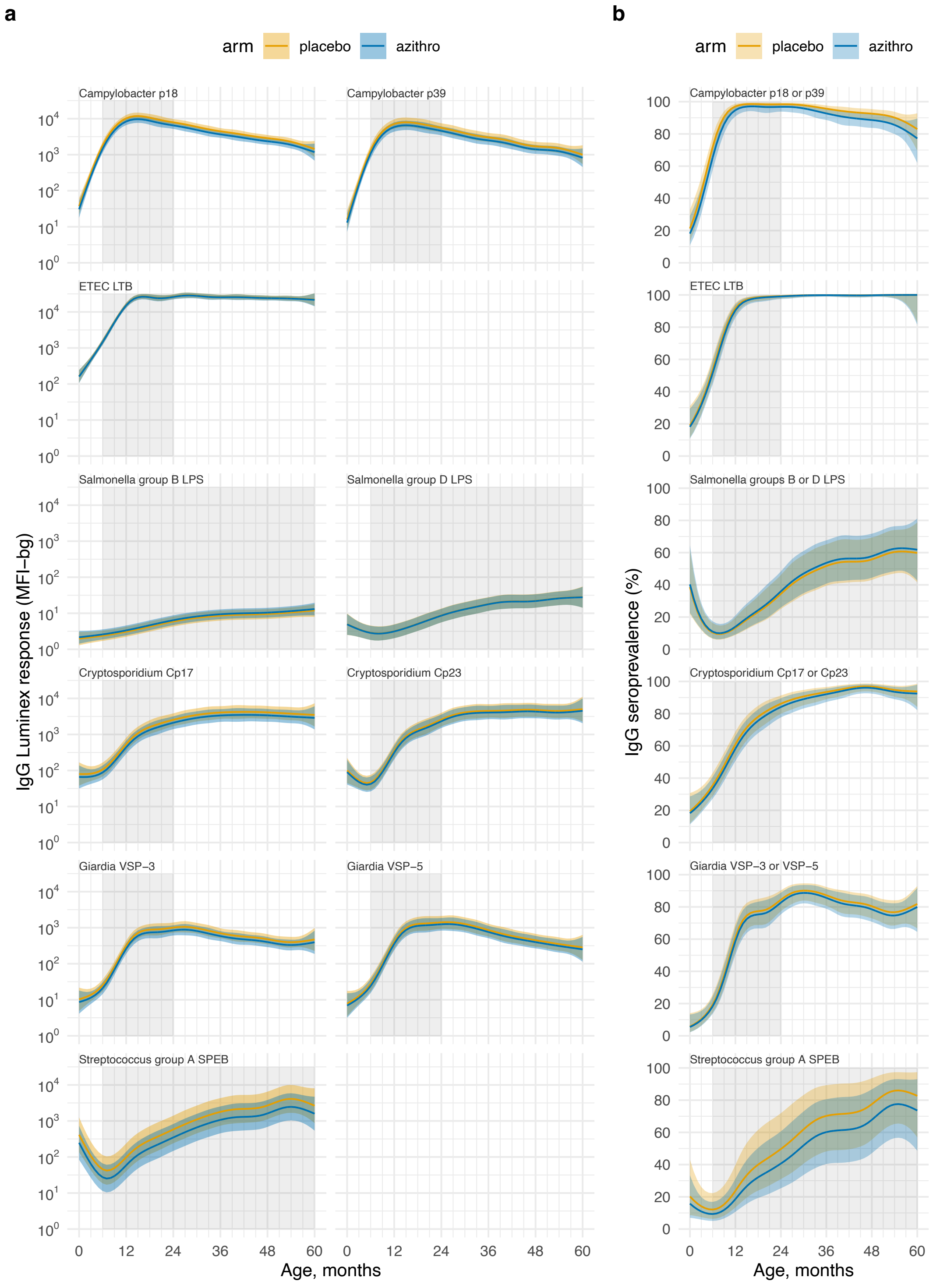

Supplementary Figure 9. **Bacterial and protozoan IgG antibody responses by age and treatment group.** Estimates derived from 5,642 measurements among children ages 1-59 months in the MORDOR Niger trial, 2015-2018 **a** Mean IgG responses (MFI-bg). **b** Seroprevalence. Group means (lines) estimated using semiparametric cubic splines. Shaded bands indicate simultaneous 95% confidence intervals. Shaded area indicates age range included in force of infection analyses (n=4,265 children ages 6-59 months, n=1,496 children ages 6-24 months). Created with notebook https://osf.io/smwbn .

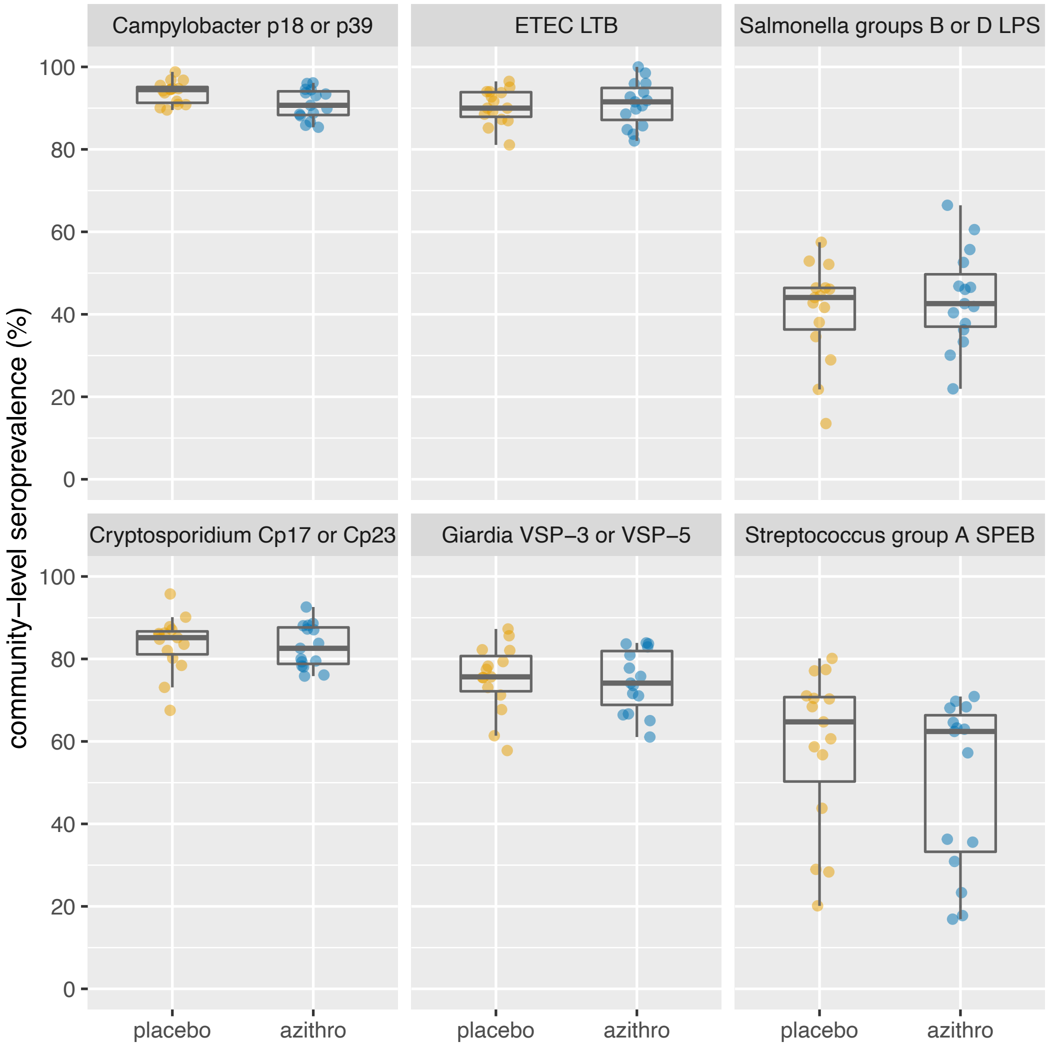

Supplementary Figure 10. Community level IgG seroprevalence for bacteria and protozoan pathogens. Estimates derived from 4,265 children ages 6-59 months in the MORDOR Niger trial, 2015-2018. Box plots indicate median (middle line) and 25th, 75th percentile (box) and 1.5 times the interquartile range (whiskers) for the 15 communities (points) in each of the treatment groups (placebo, azithromycin). Created with notebook https://osf.io/b2v3r.

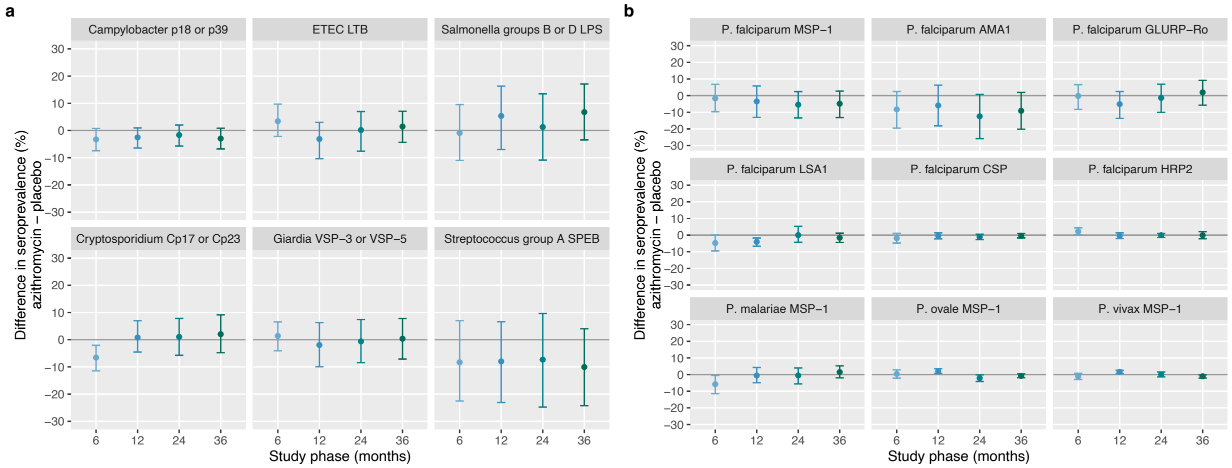

Supplementary Figure 11. Stratification by study phase. Difference in seroprevalence between intervention groups for bacterial and protozoan pathogens and malaria among children in the MORDOR Niger trial, 2015-2018, stratified by study phase (months since baseline). **a** Bacteria and protozoan antibody responses measured from 4,265 children ages 6-59 months. Points indicate mean differences between groups and error bars indicate 95% confidence intervals. **b** Malaria antibody responses measured from 3,860 children ages 12-59 months. Points indicate mean differences between groups and error bars indicate 95% confidence intervals. There was no evidence for additive effect modification by study phase. Created with notebook https://osf.io/w2rvp, which includes additional details including formal tests of effect modification on the additive scale.

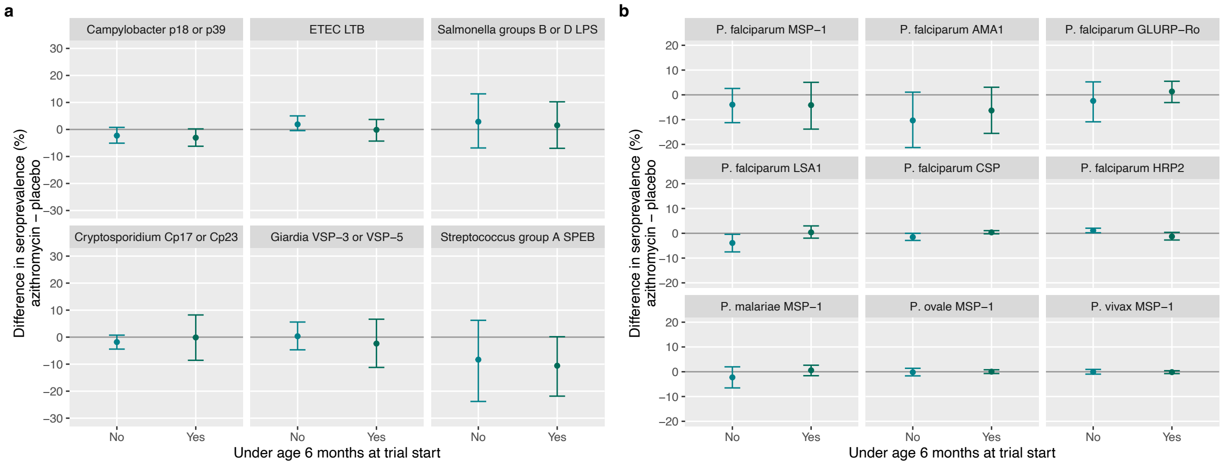

Supplementary Figure 12. Stratification by child age at trial start. Difference in IgG seroprevalence between intervention groups for bacterial and protozoan pathogens and malaria among children under age five years in MORDOR Niger, 2015-2018, stratified by child age at the start of the trial. Children who were younger than 6 months at the start of the trial may have not been born at the trial start but were enrolled in later visits. IgG responses were not included in the analysis until children were older than age 6 months (bacteria and protozoa) or 12 months (malaria) to avoid maternal IgG contributions (a pre-specified rule). **a** Bacterial and protozoan antibody responses measured from 4,265 children ages 6-59 months. Points indicate mean differences between groups and error bars indicate 95% confidence intervals. **b** Malaria antibody responses measured from 3,860 children ages 12-59 months. Points indicate mean differences between groups and error bars indicate 95% confidence intervals. There was no evidence for additive effect modification by child age at trial start. Created with notebook https://osf.io/w2rvp, which includes additional details including formal tests of effect modification on the additive scale.

Supplementary Figure 13. Community level relationship between *Plasmodium falciparum* seroprevalence and malaria parasitemia. Malaria parasitemia estimated among 4,622 children 1-59 months and seroprevalence estimated among 4,834 children ages 12-59 months (children <12 months excluded due to maternal IgG contributions, per the primary analyses) in the MORDOR Niger trial. Community means (points) are colored by intervention group. Lines represent locally weighted regression smooths, trimmed to avoid edge effects. **a** All *P. falciparum* antigens included, Spearman rank correlation ρ=0.45, exact test that $\rho \neq 0$: P=0.01. **b** longer-duration *P. falciparum* antigens, Spearman rank correlation ρ=0.44, exact test that $\rho \neq 0$: P=0.02. **c** shorter-duration antigens *P. falciparum* antigens, Spearman rank correlation ρ=0.40, exact test that $\rho \neq 0$: P=0.03. Created with notebook https://osf.io/pmg4a/.

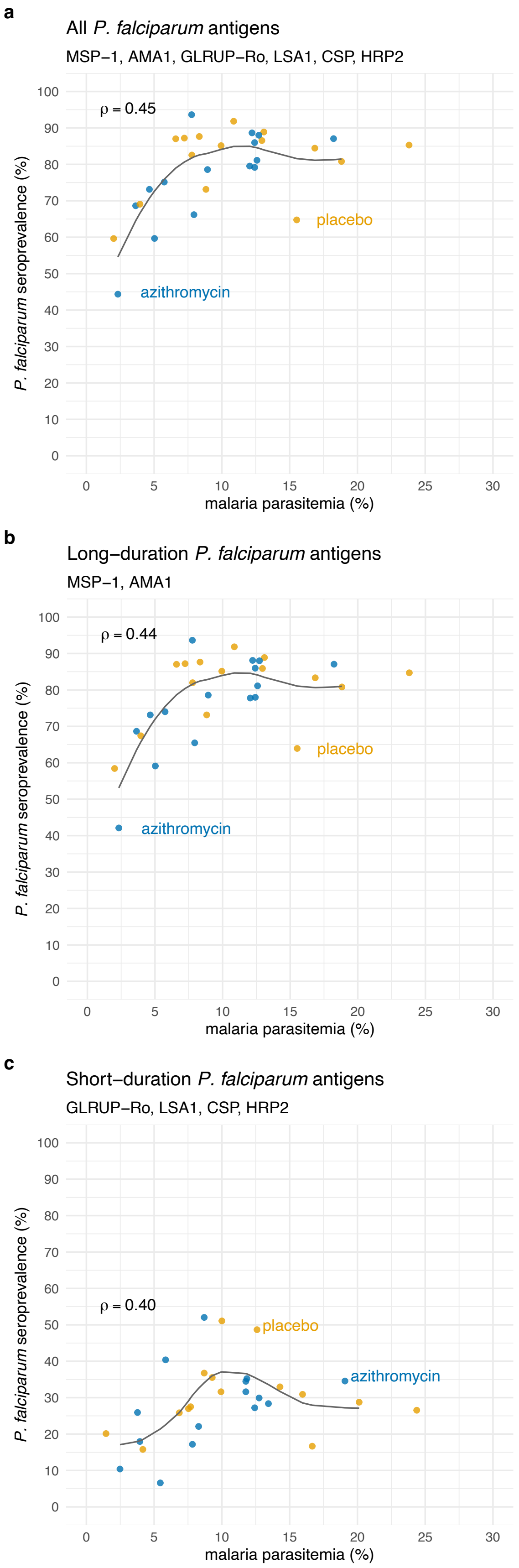

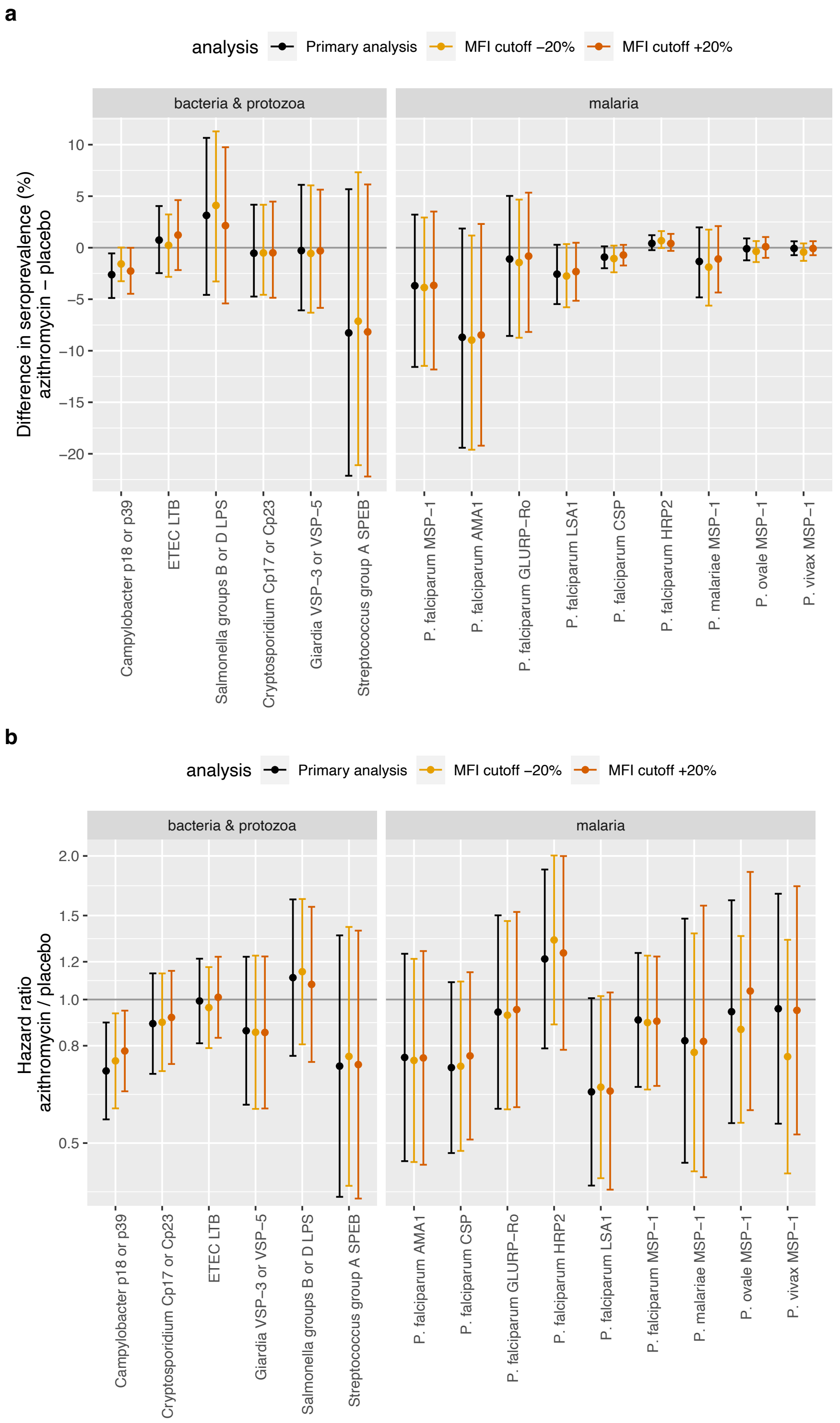

Supplementary Figure 14. Sensitivity analyses that adjusted seropositivity cutoffs by +/- 20% MFI and then repeated the primary analyses. a Difference in seroprevalence between groups. Bacterial and protozoan antibody responses measured from 4,265 children ages 6-59 months, and malarial antibody responses measured from 3,860 children ages 12-59 months. Points indicate mean differences between groups and error bars indicate 95% confidence intervals. **b** Hazard ratio of seroconversion estimated from age-structured seroprevalence. Bacterial and protozoan antibody responses measured from 1,496 children ages 6-24 months (n= 4,265 children ages 6-59 months for *Salmonella* and *Streptococcus*), and malarial antibody responses measured from 3,860 children ages 12-59 months based on pre-specified age restrictions. Points represent the hazard ratio between groups and error bars represent 95% confidence intervals. Created with notebook https://osf.io/te4vx, which additionally includes a numeric summary of cutoff changes on overall seroprevalence by antigen.

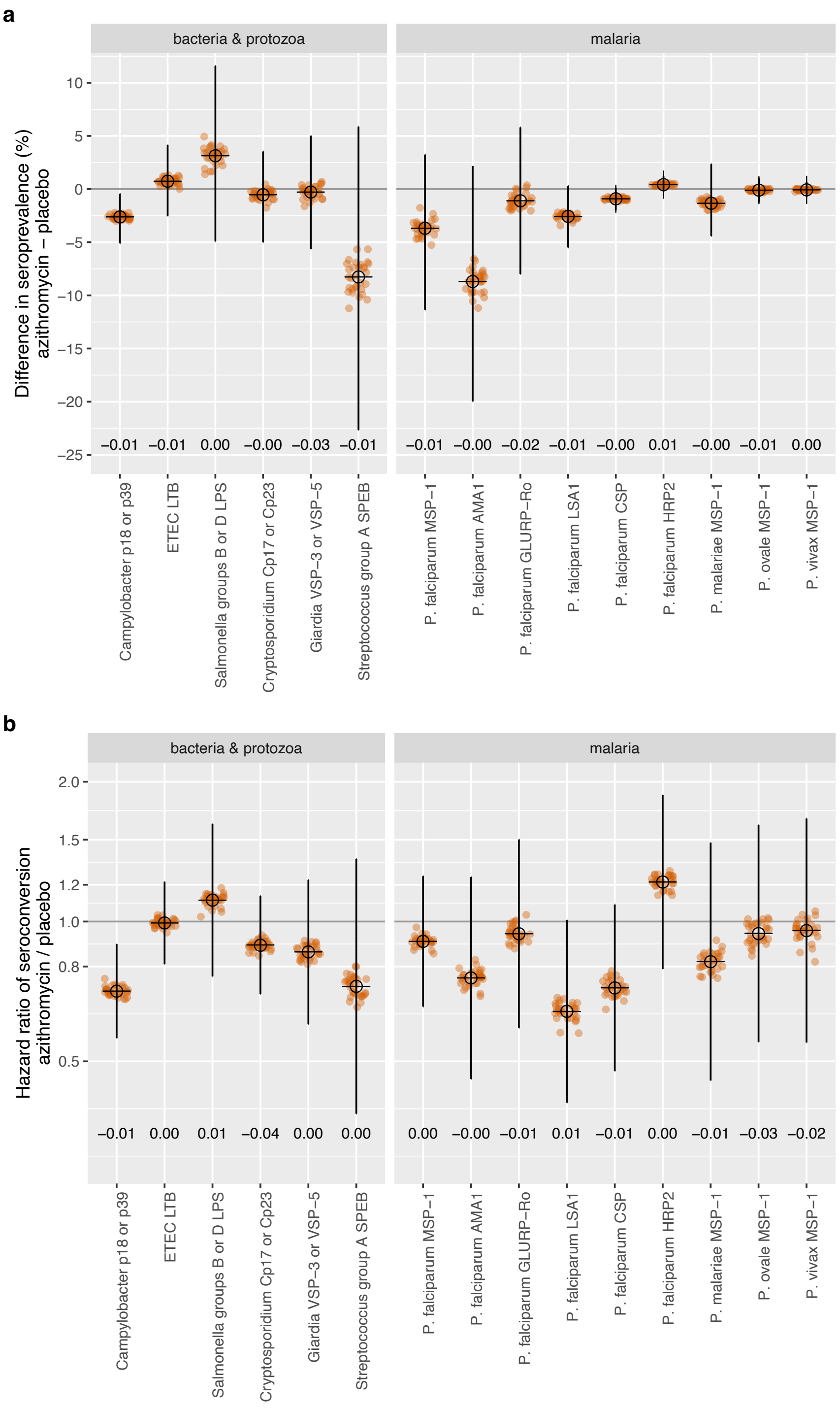

Supplementary Figure 15. **Leave-one-out sensitivity analyses.** Sensitivity analyses excluded each of the 30 study communities and re-estimated the difference in seroprevalence between groups and the relative hazard of seroconversion. Light points mark the 30 leave-one-out replicates, black circles mark the mean over leave-one-out replicates. Horizontal lines mark the mean in the full sample and vertical lines indicate the 95% confidence interval. Jackknife estimates of the bias are printed at the bottom of each panel, above the pathogen/antigen labels. All estimates are unbiased. **a** Difference in seroprevalence between groups. Bacterial and protozoan antibody responses measured from 4,265 children ages 6-59 months, and malarial antibody responses measured from 3,860 children ages 12-59 months. Jackknife estimates of the bias are on the percentage point scale, all ≤0.03%. **b** Hazard ratio of seroconversion estimated from age-structured seroprevalence. Bacterial and protozoan antibody responses measured from 1,496 children ages 6-24 months (n= 4,265 children ages 6-59 months for *Salmonella* and *Streptococcus*), and malarial antibody responses measured from 3,860 children ages 12-59 months based on pre-specified age restrictions. Jackknife estimates of the bias are on the log(hazard ratio) scale. Created with notebook https://osf.io/nvpqu.

Supplementary Table 1. Seroconversion rates for malaria, bacterial, and protozoan pathogens estimated in longitudinal analyses of children under age five years in MORDOR Niger, 2015-2018. Seroconversion was defined as transition from seronegative to seropositive status. IRR: incidence rate ratio for azithromycin / placebo seroconversion rates. Created with notebook https://osf.io/9875t.

	Azithromycin			Placebo			
**Pathogen, antigen**	**N events**	**Person-Years**	**Rate per year (95% CI)**	**N events**	**Person-Years**	**Rate per year (95% CI)**	**IRR (95% CI)**
**Malaria**							
P. falciparum MSP-1	88	173.58	0.51 (0.41, 0.65)	76	107.04	0.71 (0.47, 1.04)	0.71 (0.46, 1.19)
P. falciparum AMA1	94	345.75	0.27 (0.19, 0.38)	106	192.96	0.55 (0.40, 0.76)	0.49 (0.32, 0.78)
P. falciparum GLURP-Ro	69	606.92	0.11 (0.08, 0.15)	73	469.96	0.16 (0.11, 0.21)	0.73 (0.46, 1.14)
P. falciparum LSA1	18	684.29	0.03 (0.01, 0.06)	25	567.88	0.04 (0.02, 0.07)	0.60 (0.14, 1.56)
P. falciparum CSP	9	722.33	0.01 (0.00, 0.02)	14	588.29	0.02 (0.01, 0.03)	0.52 (0.18, 1.13)
P. falciparum HRP2	13	720.21	0.02 (0.01, 0.03)	9	596.46	0.02 (0.01, 0.02)	1.20 (0.50, 3.42)
P. malariae MSP-1	34	676.12	0.05 (0.02, 0.08)	30	563.88	0.05 (0.03, 0.07)	0.95 (0.42, 1.75)
P. ovale MSP-1	13	723.12	0.02 (0.01, 0.03)	11	602.21	0.02 (0.01, 0.03)	0.98 (0.38, 2.47)
P. vivax MSP-1	4	723.46	0.01 (0.00, 0.01)	9	604.00	0.01 (0.01, 0.02)	0.37 (0.00, 1.07)
**Bacteria & protozoa**							
Campylobacter p18 or p39	63	29.21	2.16 (1.82, 2.50)	34	12.79	2.66 (2.14, 3.22)	0.81 (0.63, 1.05)
ETEC LTB	69	33.71	2.05 (1.71, 2.50)	51	22.67	2.25 (1.85, 2.60)	0.91 (0.73, 1.20)
Salmonella groups B or D LPS	162	432.21	0.37 (0.27, 0.49)	121	403.54	0.30 (0.21, 0.39)	1.25 (0.84, 1.97)
Cryptosporidium Cp17 or Cp23	80	63.42	1.26 (0.96, 1.54)	53	45.67	1.16 (0.90, 1.48)	1.09 (0.76, 1.51)
Giardia VSP-3 or VSP-5	87	81.50	1.07 (0.81, 1.37)	53	53.04	1.00 (0.80, 1.26)	1.07 (0.73, 1.51)
Streptococcus group A SPEB	140	367.58	0.38 (0.22, 0.59)	120	288.12	0.42 (0.31, 0.60)	0.91 (0.45, 1.49)

Supplementary Table 2. Seroreversion rates for malaria, bacterial, and protozoan pathogens estimated in longitudinal analyses of children under age 5 years in MORDOR Niger, 2015-2018. Seroreversion was defined as a transition from seropositive to seronegative status. IRR: incidence rate ratio for azithromycin / placebo seroreversion rates. The IRR was not estimated for ETEC LTB because there were no seroreversion events in the placebo group. Created with notebook https://osf.io/9875t.

	Azithromycin			Placebo			
**Pathogen, antigen**	**N events**	**Person-Years**	**Rate per year (95% CI)**	**N events**	**Person-Years**	**Rate per year (95% CI)**	**IRR (95% CI)**
**Malaria**							
P. falciparum MSP-1	42	493.04	0.09 (0.06, 0.11)	35	439.25	0.08 (0.05, 0.11)	1.07 (0.67, 1.74)
P. falciparum AMA1	35	320.71	0.11 (0.07, 0.16)	38	336.12	0.11 (0.08, 0.16)	0.97 (0.53, 1.64)
P. falciparum GLURP-Ro	34	81.58	0.42 (0.29, 0.60)	28	87.71	0.32 (0.20, 0.46)	1.31 (0.80, 2.28)
P. falciparum LSA1	20	38.25	0.52 (0.35, 0.87)	21	22.92	0.92 (0.67, 1.23)	0.57 (0.34, 1.03)
P. falciparum CSP	12	9.79	1.23 (0.68, 1.58)	14	10.92	1.28 (0.90, 1.79)	0.96 (0.49, 1.46)
P. falciparum HRP2	12	9.75	1.23 (0.62, 1.82)	12	6.00	2.00 (1.30, 3.43)	0.62 (0.27, 1.13)
P. malariae MSP-1	25	38.58	0.65 (0.38, 1.10)	25	21.62	1.16 (0.71, 1.62)	0.56 (0.30, 1.13)
P. ovale MSP-1	15	8.08	1.86 (1.14, 2.75)	2	2.58	0.77 (0.00, 1.20)	2.40 (1.24, 5.03)
P. vivax MSP-1	13	9.96	1.31 (0.90, 1.90)	3	2.21	1.36 (1.20, 1.60)	0.96 (0.62, 1.42)
**Bacteria & protozoa**							
Campylobacter p18 or p39	3	103.62	0.03 (0.00, 0.06)	1	93.50	0.01 (0.00, 0.04)	2.71 (0.00, 5.49)
ETEC LTB	2	96.62	0.02 (0.00, 0.05)	0	76.33	0	n/a
Salmonella groups B or D LPS	56	298.50	0.19 (0.12, 0.27)	40	209.62	0.19 (0.14, 0.26)	0.98 (0.57, 1.65)
Cryptosporidium Cp17 or Cp23	24	55.29	0.43 (0.28, 0.64)	7	45.92	0.15 (0.05, 0.24)	2.85 (1.56, 9.95)
Giardia VSP-3 or VSP-5	11	38.92	0.28 (0.09, 0.60)	2	44.00	0.05 (0.00, 0.12)	6.22 (1.20, 23.02)
Streptococcus group A SPEB	55	388.79	0.14 (0.10, 0.20)	39	328.25	0.12 (0.06, 0.20)	1.19 (0.69, 2.58)

Supplementary Table 3. Proportion seropositive, standard deviation (SD) and community-level ICC for malarial, bacterial, and protozoan IgG responses based on *n* children in *m* communities in Niger, 2015-2018. Created with notebook https://osf.io/b2v3r/.

Antibody	m	n	Seroprevalence (SD)	ICC (95% CI)*
**Malaria †**				
P. falciparum MSP-1	30	3,860	0.75 (0.117)	0.05 (0.02, 0.09)
P. falciparum AMA1	30	3,860	0.59 (0.169)	0.11 (0.06, 0.16)
P. falciparum GLURP-Ro	30	3,860	0.20 (0.095)	0.05 (0.02, 0.08)
P. falciparum LSA1	30	3,860	0.07 (0.047)	0.02 (0.01, 0.04)
P. falciparum CSP	30	3,860	0.03 (0.016)	0.00 (0.00, 0.01)
P. falciparum HRP2	30	3,860	0.02 (0.011)	0.00 (0.00, 0.00)
P. malariae MSP-1	30	3,860	0.09 (0.048)	0.03 (0.01, 0.05)
P. ovale MSP-1	30	3,860	0.02 (0.015)	0.00 (0.00, 0.01)
P. vivax MSP-1	30	3,860	0.01 (0.010)	0.00 (0.00, 0.00)
**Bacteria and protozoa ‡**				
Campylobacter p18 or p39	30	4,265	0.93 (0.035)	0.01 (0.00, 0.02)
ETEC LTB	30	1,496	0.91 (0.047)	0.00 (0.00, 0.02)
Salmonella groups B or D LPS	30	4,265	0.43 (0.117)	0.05 (0.02, 0.08)
Cryptosporidium Cp17 or Cp23	30	4,265	0.83 (0.060)	0.02 (0.01, 0.03)
Giardia VSP-3 or VSP-5	30	4,265	0.75 (0.078)	0.02 (0.01, 0.04)
Streptococcus group A SPEB	30	4,265	0.55 (0.200)	0.15 (0.08, 0.22)

* Intra-cluster correlation coefficient (ICC). Its 95% confidence interval (CI) was estimated with a parametric bootstrap and 1000 iterations.

† Malaria antibody seroprevalence estimated among children ages 12-59 months, per the age group used in the primary analysis.

‡ Bacteria and protozoan antibody seroprevalence estimated among children ages 6-24 months (ETEC LTB) or among children ages 6-59 months (all others), per the age groups used in the primary analysis

Supplementary Table 4. Homology for *Campylobacter jejuni* p18 and p39 antigens.

Results from a Basic Local Alignment Search Tool for Proteins (BLASTP) analysis.

	p18 (cj0113/ Omp18/ PAL)				p39 (amino acids 188-508 of cj0017c/ dsbI)			
**Species**	**Strain**	**Accession No.**	**Identity (%)**	**Similarity (%)**	**Strain**	**Accession No.**	**Identity (%)**	**Similarity (%)**
Campylobacter coli	PNUSAC008160	EAH9852560	100+	100	PNUSAC008160	EAH9851771	99	99
Campylobacter lari	2003D-6133	EAI4455352	100+	100	PNUSAC009717	EBF6097769	100	100
Campylobacter upsaliensis	AG20-0002	EDP7905829	88+	94	PNUSAC003222	EAJ2130115	80	89
Campylobacter fetus	PNUSAC002822	EAK5660503	94+	98	PNUSAC008973	EAI3655429	99	99
Campylobacter hyointestinalis	PNUSAC003488	EAK5450665	100+	100	PNUSAC002793	EAI7421273	92	95
Campylobacter helveticus		WP_082199026	88+	93		WP_131937627	79	88
Campylobacter rectus		WP_124851039	56+	72		WP_004319693	71	83
Campylobacter mucosalis		WP_169764118	55w	77		WP_171993456	64	77
Campylobacter sputorum		WP_033916677	56w	73		WP_089182023	62	77
Helicobacter pylori		WP_140528957	33nr	53		WP_126442681	32	53
Haemophilus influenzae		WP_061720771	36nr	51		Not found		
Escherichia coli	NCTC13353	SQB78611	36	53		Not found		
Salmonella enterica	serovar Eastbourne	EBS5740044	37	56		Not found*		

Table Footnotes:

Results from Burnens, A., Stucki, U., Nicolet, J. & Frey, J. Identification and characterization of an immunogenic outer membrane protein of *Campylobacter jejuni*. *J. Clin. Microbiol.* **33,** 2826–2832 (1995)

+ Cross-reactive with *C. jejuni* antibody
w Weakly cross-reactive with *C. jejuni* antibody
nr Non-reactive with *C. jejuni* antibody

*Amino acids 1-187 of the dsbI protein are present in the genome, but p39 is not

Supplementary Table 5. Antigen coupling conditions used in the multiplex bead assay.

**Antigen**	**µg of antigen/ 12.5x10^6 beads**	**Coupling Buffer**
Plasmodium falciparum MSP-1_{19}	20	50 mM MES, 0.85% NaCl, pH 5
Plasmodium falciparum AMA1	20	50 mM MES, 0.85% NaCl, pH 5
Plasmodium falciparum GLURP-Ro	30	50 mM MES, 0.85% NaCl, pH 5
Plasmodium falciparum LSA1	60	50 mM MES, 0.85% NaCl, pH 5
Plasmodium falciparum CSP	30	50 mM MES, 0.85% NaCl, pH 5
Plasmodium falciparum HRP2	30	50 mM MES, 0.85% NaCl, pH 5
Plasmodium malariae MSP-1_{19}	20	50 mM MES, 0.85% NaCl, pH 5
Plasmodium ovale MSP-1_{19}	20	50 mM MES, 0.85% NaCl, pH 5
Plasmodium vivax MSP-1_{19}	20	50 mM MES, 0.85% NaCl, pH 5
Campylobacter sp. p18	25	50 mM MES, 0.85% NaCl, pH 5
Campylobacter sp. p39	25	50 mM MES, 0.85% NaCl, pH 5
ETEC LTB	30	50 mM MES, 0.85% NaCl, pH 5
Vibrio cholerae CTB	30	50 mM MES, 0.85% NaCl, pH 5
Salmonella sp. LPS serogroup B	10	50 mM MES, 0.85% NaCl, pH 5
Salmonella sp. LPS serogroup D	10	50 mM MES, 0.85% NaCl, pH 5
Cryptosporidium sp. Cp17	6.8	50 mM MES, 0.85% NaCl, pH 5
Cryptosporidium sp. Cp23	12.5	50 mM MES, 0.85% NaCl, pH 5
Giardia sp. VSP-3	60	50 mM MES, 0.85% NaCl, pH 5
Giardia sp. VSP-5	60	50 mM MES, 0.85% NaCl, pH 5
Streptococcus sp. serogroup A SPEB	60	1XPBS pH 7.2–7.4, non-ionic
