# Supplementary material for: Effect of biannual azithromycin distribution on antibody responses to malaria, bacterial, and protozoan pathogens in Niger
Source: Nat Commun. 2022 Feb 21;13:976. doi: 10.1038/s41467-022-28565-5 (PMC8861117; doi:10.1038/s41467-022-28565-5)
Supplement: Supplementary file 3 — Reporting Summary [file 41467_2022_28565_MOESM3_ESM.pdf]

nature portfolio

Corresponding author(s): Benjamin F. Arnold

Last updated by author(s): Jan 18, 2022

Reporting Summary

Nature Portfolio wishes to improve the reproducibility of the work that we publish. This form provides structure for consistency and transparency in reporting. For further information on Nature Portfolio policies, see our Editorial Policies and the Editorial Policy Checklist.

Statistics

For all statistical analyses, confirm that the following items are present in the figure legend, table legend, main text, or Methods section.

n/a	Confirmed	
☐	☒	The exact sample size (*n*) for each experimental group/condition, given as a discrete number and unit of measurement
☐	☒	A statement on whether measurements were taken from distinct samples or whether the same sample was measured repeatedly
☐	☒	The statistical test(s) used AND whether they are one- or two-sided<br>*Only common tests should be described solely by name; describe more complex techniques in the Methods section.*
☐	☒	A description of all covariates tested
☐	☒	A description of any assumptions or corrections, such as tests of normality and adjustment for multiple comparisons
☐	☒	A full description of the statistical parameters including central tendency (e.g. means) or other basic estimates (e.g. regression coefficient) AND variation (e.g. standard deviation) or associated estimates of uncertainty (e.g. confidence intervals)
☐	☒	For null hypothesis testing, the test statistic (e.g. *F*, *t*, *r*) with confidence intervals, effect sizes, degrees of freedom and *P* value noted<br>*Give P values as exact values whenever suitable.*
☒	☐	For Bayesian analysis, information on the choice of priors and Markov chain Monte Carlo settings
☐	☒	For hierarchical and complex designs, identification of the appropriate level for tests and full reporting of outcomes
☐	☒	Estimates of effect sizes (e.g. Cohen's *d*, Pearson's *r*), indicating how they were calculated

Our web collection on statistics for biologists contains articles on many of the points above.

Software and code

Policy information about availability of computer code

Data collection	Field data were collected using handheld tablets (Android operating system version 5) and a custom application designed for the study (versions 2-4, Conexus Inc., Los Gatos, CA), which encrypted and transmitted the data to a secure server hosted by Salesforce.com.
Data analysis	All code used in the study is available through GitHub (https://github.com/proctor-ucsf/mordor-antibody) and the Open Science Framework (https://osf.io/954bt). Analyses used R statistical software, version 4.1.1. The study's GitHub site includes package details.

For manuscripts utilizing custom algorithms or software that are central to the research but not yet described in published literature, software must be made available to editors and reviewers. We strongly encourage code deposition in a community repository (e.g. GitHub). See the Nature Portfolio guidelines for submitting code & software for further information.

Data

Policy information about availability of data

All manuscripts must include a data availability statement. This statement should provide the following information, where applicable:

- Accession codes, unique identifiers, or web links for publicly available datasets
- A description of any restrictions on data availability
- For clinical datasets or third party data, please ensure that the statement adheres to our policy

De-identified individual participant data generated in this study have been deposited in the Open Science Framework (DOI: https://osf.io/954bt) and in the Dryad database (DOI: https://doi.org/10.7272/Q6VX0DSD). Results from BLASTP searches, including accession numbers, are summarized in Supplementary Table 4.

March 2021

Field-specific reporting

Please select the one below that is the best fit for your research. If you are not sure, read the appropriate sections before making your selection.

☒ Life sciences ☐ Behavioural & social sciences ☐ Ecological, evolutionary & environmental sciences

For a reference copy of the document with all sections, see nature.com/documents/nr-reporting-summary-flat.pdf

Life sciences study design

All studies must disclose on these points even when the disclosure is negative.

Sample size	This text is from the substudy's publicly posted Statistical Analysis Plan (https://osf.io/d9s4t/). The MORDOR morbidity monitoring trial was designed around the primary antimicrobial resistance monitoring endpoints. We informed the sample size calculation for the antibody substudy analysis with measurements from the PRET trial, where 991 children ages 1-5 years old were measured for antibody response to Plasmodium falciparum MSP-119 across 24 communities. In that study, mean seroprevalence to MSP-1 was 65% and the intra-class correlation coefficient for seropositivity was 0.004. We used a standard sample size equation for cluster randomized trials in binary outcomes (equation 7.11 of Hayes and Moulton 2017). Under these assumptions, we estimated that with 15 communities per arm, 140 measurements per community (four phases) and 80% power, the minimum detectable relative reduction is 8% (prevalence difference = –5.4 percentage points). At 90% power, the detectable relative reduction is 10% (prevalence difference = –6.3 percentage points). We estimated under the same assumptions that with 35 children per community (single phase), at 80% power the minimum detectable relative reduction is 14% (prevalence difference = –9.3 percentage points). At 90% power the detectable relative reduction is 16% (prevalence difference = –10.7 percentage points).
Data exclusions	Data were excluded from children 1-12 months old (malaria analyses) and 1-6 months old (bacteria and protozoa analyses) to remove potential contributions from maternal IgG. These exclusions were based on a pre-specified rule in the Statistical Analysis Plan.
Replication	Samples were run on 63 plates and each plate included positive controls from a high positive sera pool (1:400 dilution), a low positive sera pool (1:6400 dilution) and a normal human sera (single) control. To assess plate-to-plate variation, we estimated the standard deviation (SD) for each antigen's responses across plates. The laboratory protocol specified that a plate should be re-analyzed if more than half of the antigens had SDs that deviated by >20% from the overall average in multiple controls. Two of 63 plates failed this criterion for one control but passed in the other two controls and were thus not repeated. For the positive control sample responses to the 22 antigens used in this study, the average CV% was 10.0 with a standard deviation of 4.9. The median CV% was 9.3 with a range of 2.9% to 20.2%. These values were deemed sufficient to meet our criteria for internal replication of laboratory analyses.<br><br>Statistical analyses were not internally replicated, but we have provided source data and code needed to replicate all of the analyses. They are available through the Open Science Framework (https://osf.io/954bt/) and cross-listed with GitHub and Dryad (links through the OSF project).
Randomization	Communities were randomized 1:1 using a sequence the trial biostatistician generated.
Blinding	Placebo and azithromycin had identical packaging to maintain masking. Unmasked members of the data team and Pfizer labeled the study drugs. Participants, field staff, laboratory staff, analysts, and all investigators were masked to treatment assignments throughout the trial. Blood samples were masked and randomly ordered by the UCSF trial coordinating center before sending them to the laboratory at the CDC. Masked analyses were completed at UCSF using a shuffled version of the treatment assignment variable. Data were unmasked only after the final table and figure shells had been populated (documented through the article's GitHub repository).

Reporting for specific materials, systems and methods

We require information from authors about some types of materials, experimental systems and methods used in many studies. Here, indicate whether each material, system or method listed is relevant to your study. If you are not sure if a list item applies to your research, read the appropriate section before selecting a response.

Materials & experimental systems

n/a	Involved in the study	
☐	☒	Antibodies
☒	☐	Eukaryotic cell lines
☒	☐	Palaeontology and archaeology
☒	☐	Animals and other organisms
☐	☒	Human research participants
☐	☒	Clinical data
☒	☐	Dual use research of concern

Methods

n/a	Involved in the study	
☒	☐	ChIP-seq
☒	☐	Flow cytometry
☒	☐	MRI-based neuroimaging

Antibodies

Antibodies used	Mouse anti-human IgG Fc-Biot (supplier: Southern Biotech; catalog number:

	Streptavidin, R-Phycoerythrin Conjugate (SAPE) (supplier: Invitrogen; catalog number: S-866; Clone: n/a, Lot number: 2044866; Stock concentration: 1 mg/ml; ul/5.5 mL Buffer: 27.5; Antibody dilution: 1:200; ng/well: 250).
Validation	All antibodies have been validated for use with human sera in ELISA, FLISA, and multiplex assays as documented on their technical bulletins on the suppliers' websites. https://www.southernbiotech.com/techbul/9042.pdf https://www.southernbiotech.com/techbul/9200.pdf https://www.thermofisher.com/order/catalog/product/S866

Human research participants

Policy information about studies involving human research participants

Population characteristics	The study enrolled 3,814 children aged 1-59 months and tested a total of 5,642 blood specimens through the 36-month follow-up between March 2015 and June 2018. 46% of enrolled children were female, 12% were <1 year old and 27% were <2 years old. At enrollment, IgG antibody testing showed evidence for high levels of previous exposure to malaria and bacterial and protozoan enteric pathogens (details in Table 1 of the manuscript).
Recruitment	MORDOR Niger was a cluster-randomized, placebo-controlled trial that randomized at the community level because of the intervention's campaign-style, biannual mass distribution. Communities with 200 to 2,000 inhabitants based on the Niger 2012 census were eligible for inclusion in the trial, and children ages 1–59 months who weighed >3.8 kg were eligible for treatment.
Ethics oversight	The trial protocol was reviewed and approved by the Committee on Human Research at the University of California, San Francisco, and the Niger Ministry of Health's Ethical Committee. An independent Data and Safety Monitoring Committee provided additional oversight.

Note that full information on the approval of the study protocol must also be provided in the manuscript.

Clinical data

Policy information about clinical studies

All manuscripts should comply with the ICMJEguidelines for publication of clinical research and a completedCONSORT checklist must be included with all submissions.

Clinical trial registration	NCT02048007
Study protocol	Supplementary Information File of Doan et al. Nature Medicine. 25, 1370–1376 (2019) https://www.nature.com/articles/s41591- 019-0533-0?proof=tr+#Sec15
Data collection	A total of 5,642 blood specimens were collected from 3,814 enrolled children aged 1-59 months in the 30 study communities in the Dosso region of Niger between March 2015 and June 2018. Dried fingerprick blood spots (DBS) were collected onto calibrated filter paper wheels with 6 10µl extensions (TropBio Pty Ltd., Townsville, Queensland, Australia) by mobile field teams in the rural, Nigerien communities.
Outcomes	Antibody-based measures of previous infection were pre-specified secondary outcomes for the trial, specified on clinicaltrials.gov (NCT02048007). Additionally, before accessing any antibody testing results, we prespecified all analyses in the antibody substudy's Statistical Analysis Plan, time-stamped and archived through the Open Science Framework (https://osf.io/d9s4t/).

March 2021
